# DNS-Sensor: A Sensor-Driven Architecture for Real-Time DNS Cache Poisoning Detection and Mitigation

**DOI:** 10.3390/s25226884

**Published:** 2025-11-11

**Authors:** Haisheng Yu, Xuebiao Yuchi, Xue Yang, Hongtao Li, Xingxing Yang, Wei Wang

**Affiliations:** 1 China Internet Network Information Center, Beijing 100190, China; 2 Saiyu Technology Co., Ltd., Beijing 100190, China

**Keywords:** domain name system, cache poisoning, cache consistency checking, disaster resolution, fragmentation attack

## Abstract

The Domain Name System (DNS) is a fundamental component of the Internet, yet its distributed and caching nature makes it susceptible to various attacks, especially cache poisoning. Although the use of random port numbers and transaction IDs has reduced the probability of cache poisoning, recent developments such as DNS Forwarder fragmentation and side-channel attacks have increased the possibility of cache poisoning. To counteract these emerging cache poisoning techniques, this paper proposes the DNS Cache Sensor (DNS-Sensor) system, which operates as a distributed sensor network for DNS security. Like environmental sensors monitoring physical parameters, DNS-Sensor continuously scans DNS cache records, comparing them with authoritative data to detect anomalies with sensor-grade precision. It involves checking whether the DNS cache is consistent with authoritative query results by continuous observation to determine whether cache poisoning has occurred. In the event of cache poisoning, the system switches to a disaster recovery resolution system. To expedite comparison and DNS query speeds and isolate the impact of cache poisoning on the disaster recovery resolution system, this paper uses a local top-level domain authoritative mirror query system. Experimental results demonstrate the accuracy of the DNS-Sensor system in detecting cache poisoning, while the local authoritative mirror query system significantly improves the efficiency of DNS-Sensor. Compared to traditional DNS, the integrated DNS query and DNS-Sensor method and local top-level domain authoritative mirror query system is faster, thus improving DNS performance and security.

## 1. Introduction

Serving as the global Internet’s address book, the Domain Name System (DNS) provides the mapping functionality between human-friendly domain names and machine-readable IP addresses [1]. As such, the implementation of almost all kinds of Internet-based activities is based on the DNS. Due to the explosive growth of the Internet, the DNS has suffered rapid expansions over the decades (such as strong volume growth in terms of namespace, nameservers, and network traffic), leading to today’s most critical and successful distributed system in the world.

At the same time, due to its openness and distributed nature, the DNS system faces various security threats, of which DNS cache poisoning attacks are one of the most notorious [2]. These attacks not only disrupt the normal operation of the Internet but can also lead to hazards such as user data leakage, hijacking of sensitive information, and malicious tampering of website content. The notorious Kaminsky [3] attack raised global awareness of the severity of DNS cache poisoning and prompted the deployment of several protocol-level defense mechanisms. Despite existing methods and technologies, such as the DNSSEC protocol, firewalls, and intrusion detection systems being widely used to detect and mitigate DNS cache poisoning, a series of new vulnerabilities [4,5,6] in recent years have reignited the threat of cache poisoning and DNS hijacking. In particular, the fragment assembly attack against DNS Forwarders is a relatively new and more challenging attack method [7]. It exploits the fragmentation feature of DNS response packets to bypass existing defense measures, caching malicious response data into DNS Forwarders, thereby causing cache poisoning. This type of attack poses a threat not only to ordinary users but also to cybersecurity of enterprises and organizations.

To effectively counter this emerging threat, this paper proposes a novel defense method, the DNS Cache Sensor (DNS-Sensor) system. The DNS-Sensor system operates as a distributed digital sensor network, constantly monitoring the DNS cache for any signs of cache poisoning. By regularly checking the consistency between the cache and authoritative query results, it can promptly detect and prevent cache poisoning, similar to how a sensor detects deviations from normal environmental parameters. In comparison to traditional methods, the DNS-Sensor system does not rely on the deployment of DNSSEC, nor does it require truncation or rewriting of response data, thereby reducing additional computation and bandwidth overhead.

This paper provides a detailed explanation of the principles and implementation of the DNS-Sensor system and evaluates its performance in an experimental environment. Through comparative analysis of experimental results, it validates the effectiveness and feasibility of the proposed method. Furthermore, this paper delves into the advantages of the DNS-Sensor system, elucidating its potential application in mitigating fragment assembly attacks against DNS Forwarders. The DNS-Sensor system establishes a sensor–actuator framework for DNS protection. Its sensing module performs continuous surveillance of cache records, while its actuation module triggers disaster recovery when threats are detected—mirroring how industrial sensor systems respond to hazardous conditions. This closed-loop design enables sub-second detection and mitigation of cache poisoning attacks.

## 2. Background and Related Work

### 2.1. DNS System Overview

**Figure 1:**
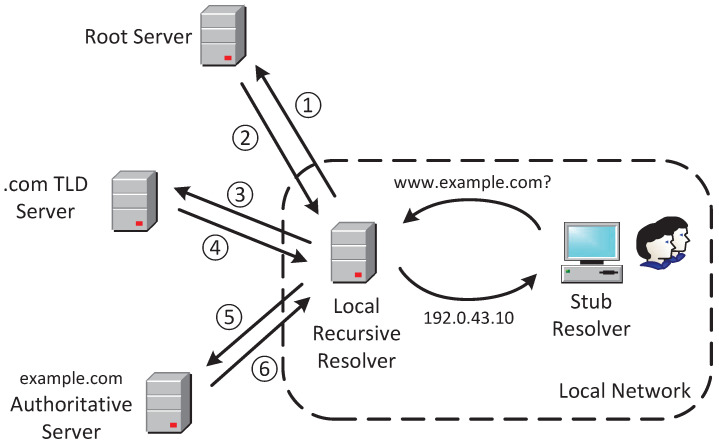
Standard DNS resolution architecture and flow.

The Domain Name System (DNS) [8] is a distributed and hierarchical naming system that translates domain names into IP addresses. When a client device, known as a stub resolver, needs to resolve a domain such as www.example.com, it sends a query to a recursive resolver, which is typically managed by the local network administrator but can also be an external service like Google Public DNS or OpenDNS. If the recursive resolver has no cached record for the requested domain (or the existing record’s Time-to-Live (TTL) has expired), it begins the resolution process by querying the Root DNS server. The Root server responds with a referral to the appropriate Top-Level Domain (TLD) server, in this case, the .com TLD server. The recursive resolver then queries the .com TLD server, which in turn refers it to the authoritative DNS server for example.com. Finally, the recursive resolver contacts the authoritative server for example.com. The authoritative server provides the corresponding IP address for www.example.com along with a TTL value that specifies how long the resolver should cache this record before considering it stale. This step completes the resolution process. Figure 1 illustrates this process.

### 2.2. Background

DNS cache poisoning attacks pose a significant challenge in the current landscape of internet security. Numerous researchers and security experts have proposed various methods and technologies to counter this threat. One prevalent attack type is the spoofing attack, where the goal is to craft a malicious DNS response and deceive the resolver into accepting it [9]. Attackers manipulate fields in forged packets to match DNS queries, such as the question section, DNS Transaction ID, source/destination addresses, and port numbers. The 2008 Kaminsky [3] attack is one of the most impactful instances of a spoofing attack, influencing nearly all DNS software designs. To mitigate the success rate of spoofing attacks, existing defense measures include increasing the randomness of DNS query packets, such as using random temporary port numbers and DNS transaction IDs, and employing 0x20 encoding to mix the case spelling of names in the query.

Another type of attack targeting the DNS cache is the fragment assembly attack, exploiting the fragmentation of DNS response packets to circumvent randomized spoofing attack defenses. Attackers create malicious DNS records, send DNS queries for the victim domain [10], and force the response from the authoritative server to be fragmented. On the recursive resolver, the attacker reassembles a legitimate first fragment with a malicious second fragment, generating a malicious DNS response and successfully poisoning the cache [11]. Two methods have been proposed to address fragment assembly attacks: PMTU-based fragment assembly attacks, which reduce the maximum transmission unit (MTU) between recursive resolvers and authoritative servers, and DNSSEC-based fragment assembly attacks, which send DNSSEC queries to enlarge the DNS response size and cause fragmentation.

While the success probability of traditional DNS cache poisoning attacks has decreased with the introduction of randomized ports and Transaction IDs [12], recent techniques such as fragment assembly attacks against DNS Forwarders [7] and port detection through ICMP packet probing [4] have significantly increased this risk.

**Figure 2:**
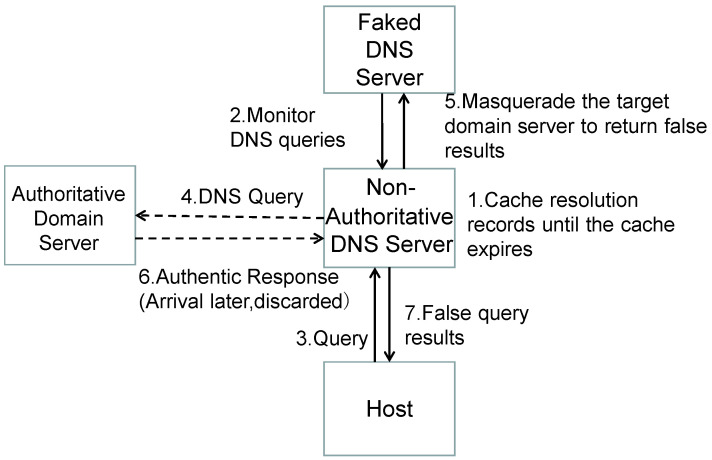
Principle of DNS cache poisoning attack.

As shown in Figure 2, the specific steps of a fragment assembly attack against DNS Forwarders are as follows:

Predict IPID: Attackers need to predict the IP Identification (IPID) of IP packets used by the target DNS Forwarder. This is crucial for the attack. Attackers can predict the next IPID by sending multiple probing packets and observing the pattern of IPID changes.

Construct the Second Fragment: Attackers construct the second fragment of the response data packet, containing a forged A record mapping the target domain to a malicious IP address. This second fragment is incomplete, lacking DNS headers and the query section, only containing response data.

Initiate DNS Query Request: Attackers initiate a DNS query request for the target domain (e.g., http://a.attacker.com). The DNS Forwarder forwards this query to the upstream recursive resolver.

Malicious Response from Authoritative Resolver: Since the authoritative domain resolver is controlled by the attacker, it returns a response data packet to the recursive resolver with numerous CNAME records, making the response data packet very large.

Fragment Packet Reassembly: Upon receiving the response data packet, the recursive resolver sends it to the DNS Forwarder in fragments. When the DNS Forwarder receives the first fragment, it reassembles it with the previously cached second fragment into a complete data packet. This reassembly is possible because the IPID of the second fragment has been predicted and matches the legitimate first fragment. As the DNS Forwarder does not perform packet inspection, it forwards this result to the DNS client.

DNS Cache Poisoning: The DNS client, upon receiving the malicious response, caches the malicious IP address. Subsequent queries for the same domain will return the malicious IP address, leading to DNS cache poisoning.

### 2.3. Related Work

To prevent DNS cache poisoning, various methods are commonly employed:

DNSSEC (Domain Name System Security Extensions): A widely used security extension mechanism that adds digital signatures to DNS packets to verify the authenticity of response data, preventing malicious data tampering. While effective, DNSSEC deployment is complex and requires support from domain registrars and DNS servers, resulting in a relatively low deployment rate in practice.

Firewalls and Intrusion Detection Systems: These are common network security devices used to monitor and filter network traffic, preventing unauthorized access and attacks [13,14]. Firewalls can be configured to limit access to DNS servers, preventing some malicious requests from entering. Intrusion detection systems identify abnormal DNS traffic and behavior, detecting potential cache poisoning attacks [15]. However, they may struggle to recognize fragment assembly attacks against DNS Forwarders, as these attacks exploit packet fragmentation characteristics, bypassing packet integrity checks.

DNS Cookies: A technology introduced in RFC7873 to address DNS security issues. It aims to counter cache poisoning and deceptive DNS amplification attacks not resolved by DNSSEC. DNS Cookies are simple to implement and offer security similar to using TCP [16], making them easier to deploy compared to DNSSEC. Clients send DNS requests with a cookie option containing the hash value of the IP address and secret. Servers use the client’s IP and secret to validate the cookie, ensuring consistency. DNS Cookies enhance DNS security through a straightforward mechanism, providing an effective supplement for defending against cache poisoning attacks. Research indicates that less than 30% of servers and less than 10% of recursive clients use DNS Cookies [17,18].

DNS Request Rate Limiting: An effective method to prevent DNS cache poisoning by restricting the query frequency from specific IP addresses within a defined timeframe. This prevents an overload of DNS servers and cache poisoning resulting from a large number of malicious requests. However, against fragment assembly attacks, attackers can bypass request rate limits by controlling legitimate DNS servers for cache poisoning.

DNS-Sensor system: A novel strategy proposed in this paper to prevent DNS cache poisoning. It conducts cache consistency checks on the response data in DNS caches, including continuous collection of DNS cache and authoritative response data from the DNS cache and authoritative sources, real-time comparison of DNS responses with authoritative query results, and promptly identifying cache poisoning, to provide an early-phase warning for potential DNS security threats.

Overall, numerous methods and technologies are available for preventing DNS cache poisoning, each with its advantages and limitations. By combining multiple methods and incorporating innovative approaches such as the DNS-Sensor system, we can build a more robust and effective DNS cache poisoning prevention system, providing reliable network security for internet users and organizations. Continuous exploration and innovation are essential to maintaining focus on internet security, continuously refining DNS cache poisoning defense strategies.

## 3. Architecture and Implementation of the DNS-Sensor System

The proposed DNS-Sensor system is an innovative strategy for preventing DNS cache poisoning. Similar to a sensor that continuously generates observed data from the environment, this method primarily involves conducting cache consistency checks on the response data in DNS caches, including continuous collection of DNS cache and authoritative response data from the DNS cache and authoritative sources, real-time comparison of DNS responses with authoritative query results, and promptly identifying cache poisoning to provide an early-phase warning for potential DNS security threats. In this section, there will be a detailed explanation of the principles and implementation of the DNS-Sensor system.

### 3.1. Principles of the DNS-Sensor System

The core principle of the DNS-Sensor system is to monitor and compare the cache data of DNS forwarders. In a standard DNS response, a DNS cache server stores response data containing IP address records related to domain names. This allows for a quick response when the same domain is queried again, reducing DNS resolution time. However, in the case of a DNS forwarder facing fragmentation attack, malicious response data may be cached, leading to cache poisoning.

The DNS-Sensor system initially establishes a cache database in the DNS forwarder, recording all cached domain names and their corresponding IP address records. It concurrently continuously validates authoritative query responses against cached records. Upon receiving a DNS query request from a user, the DNS forwarder checks the cache database. If the query result exists in the cache, it is directly returned to the user. If not, the forwarder initiates a query request to the authoritative domain resolver.

Upon receiving the response from the authoritative domain resolver, the DNS-Sensor system compares it with the cached data corresponding to the domain name in the cache database. If the IP address records match, indicating a legitimate response without cache poisoning, the DNS forwarder caches the response and returns it to the user. If a discrepancy is detected between the IP address records, a possible cache poisoning attack is identified. In such cases, the DNS-Sensor system triggers an immediate alert, ceases caching malicious response data, ensuring user safety.

As shown in Table 1, several cache comparison methods are employed, including the following:

Hash Verification Method: Computes hashes of data in both DNS cache and authoritative query cache, comparing the resulting hash values. Any inconsistency is considered a potential cache poisoning event.

Record Comparison Method: Compares the resolved results of DNS recursive cache with those returned by authoritative queries, detecting inconsistencies in resolution to identify possible cache poisoning.

Blockchain Technology: Leveraging the immutability and distributed nature of blockchain, DNS queries and cache update records are stored on the blockchain. Consistency of the cache is verified by comparing data on the blockchain [19].

Redundant Data Comparison: DNS query result are simultaneously cached in multiple independent DNS cache systems. These cached data are then compared, and any inconsistencies indicate a potential cache poisoning.

Sensor-like Properties of the DNS-Sensor System: The DNS-Sensor system embodies key characteristics of a sensor system:

Data Acquisition: It collects inputs from both cached and authoritative DNS records, analogous to a sensor sampling physical parameters.

Anomaly Detection: By comparing these inputs, it identifies deviations (e.g., mismatched IP records) with precision similar to sensor threshold alerts.

Response Mechanism: Upon detecting poisoning, it initiates countermeasures (e.g., switching to disaster recovery), paralleling how sensors trigger actuators in control systems. This framework positions the DNS-Sensor system as a cyber–physical sensor tailored for DNS ecosystems.

### 3.2. Implementation of the DNS-Sensor System

To address system complexity and computational challenges, a combination of hash verification and record comparison methods is employed for the DNS-Sensor system. The implementation involves the following steps:

Establishment of a Cache Database:

A cache database is created to record all cached domain names and their corresponding IP address records. This database can be stored using a relational or NoSQL database. Monitoring DNS Query Requests:

When the cache server receives a DNS query request from a user, it checks the cache database. If the result is present in the cache, it is directly returned to the user. Otherwise, a query request is sent to the authoritative domain resolver. Monitoring Authoritative Query Responses:

Upon receiving the response from the authoritative domain resolver, the DNS-Sensor system compares it with the cached data in the database. Hash verification and record comparison methods ensure data integrity and consistency. Cache Data Update and Alerts:

If the IP address records match, indicating a legitimate response without cache poisoning, the cache server caches the response and returns it to the user. If a discrepancy is detected, a possible cache poisoning is alerted, and caching of malicious response data is stopped. Cache Consistency Maintenance:

Regular updates and cleaning of data in the cache database are performed to ensure accuracy and consistency. A cache expiration mechanism is introduced to automatically remove expired cache data based on Time-To-Live (TTL). Through these steps, the DNS-Sensor system effectively prevents cache poisoning attacks, such as fragmentation attacks on DNS forwarders and side-channel attacks, ensuring the security and stability of DNS resolution.

### 3.3. Advantages and Limitations of the DNS-Sensor System

Advantages: Continuous validation mechanism ensuring DNS response-authoritative data consistency results. Flexibility and scalability during implementation, allowing the selection of different comparison methods and data storage based on practical considerations. Integration potential with other DNS cache poisoning prevention strategies for a comprehensive defense system, enhancing DNS resolution security.

Limitations: Real-time monitoring introduces additional computation and storage overhead, potentially impacting DNS resolution performance. Inability to prevent attacks if the authoritative server is compromised. Timely updates and maintenance of the cache database are critical to maintaining consistency, requiring accuracy and timeliness.

In Section 4, to address these limitations, we enhance the efficiency of DNS-Sensor comparison by deploying a local top-level domain authoritative mirror query system, simultaneously improving the security of the disaster recovery resolution system.

## 4. Disaster Recovery Resolution System and Optimization to Counter DNS Cache Poisoning

**Figure 3:**
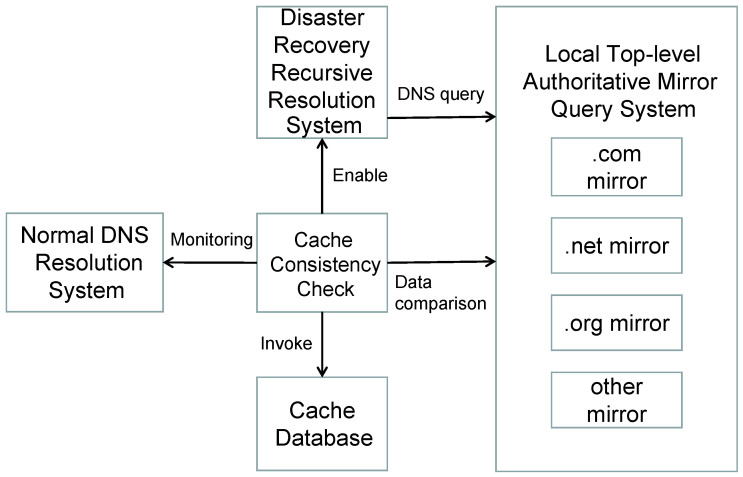
DNS-Sensor System Architecture.

In this study, we employ the DNS-Sensor system to prevent cache poisoning attacks. However, the DNS-Sensor system faces a significant performance bottleneck, requiring frequent comparisons between local cache resolution records and records obtained through authoritative queries. The query for authoritative resolution records is a time-consuming process [20,21]. To enhance the efficiency of the DNS-Sensor system and concurrently initiate a disaster recovery recursive resolution system, we implement a local top-level authoritative mirror query system to accelerate the comparison speed. The disaster recovery resolution system provides effective measures in response to detected DNS cache poisoning. This system comprises two main components, namely, the Emergency Cache Query System and the Local Top-Level Authoritative Mirror Query System, ensuring secure and reliable DNS resolution services (Figure 3).

Firstly, we introduce the Emergency Cache Query System, a set of backup servers pre-loaded with validated DNS query caches that are free from cache poisoning. When the DNS-Sensor system identifies DNS cache poisoning, indicating a discrepancy between response data in the cache and authoritative data, it promptly shifts the inconsistent DNS queries to the disaster recovery resolution servers. This ensures timely responses to user DNS queries, mitigating the adverse effects of cache poisoning on DNS resolution services.

Secondly, we incorporate the Local Top-Level Authoritative Mirror Query System into the disaster recovery resolution system. This system downloads data from the top-level domain, such as .com and .net, from the Centralized Zone Data Service (CZDS) [22], records download speeds and required time, and loads these data into a local mirrored cache. This accelerates the retrieval of authoritative data for top-level domains during DNS resolution, expediting the comparison process between cached data and authoritative data. Through this optimization measure, we successfully reduce the latency in comparing cache data and authoritative data, enhancing the overall performance of the system.

In conclusion, by introducing the Emergency Cache Query System and the Local Top-Level Authoritative Mirror Query System, DNS-Sensor effectively prevents DNS cache poisoning attacks and optimizes the data comparison speed within the DNS-Sensor system. These measures provide a robust guarantee for the security and performance of DNS resolution services, ensuring that users experience reliable and rapid resolution. Nevertheless, as attack techniques evolve, continuous improvement and optimization of defense measures are necessary to counter potential new types of DNS cache poisoning attacks. Therefore, we look forward to future research directions and remain committed to improving the security and performance of DNS systems.

## 5. Experiments and Evaluation

To validate the effectiveness and performance of the DNS-Sensor system, we conducted a series of experiments and thoroughly evaluated their results. In these experiments, fragment rearrangement attacks were simulated against DNS forwarders and the DNS-Sensor system was employed for defense. Below are the specific steps of the experiments and the evaluation results.

### 5.1. Experimental Steps

We established a simulated DNS resolution environment for the experiments, including DNS forwarders, authoritative domain name resolvers, and DNS clients. We used open-source software Bind and Unbound to set up DNS servers, deploying these servers in the local network. The .net and .com data refer to the respective zone data for the top level domains of .net and .com obtained from the Centralized Zone Data Service (CZDS).

To simulate fragment rearrangement attacks, we utilized Python scripts to construct malicious DNS response packets and sent these packets to DNS Forwarders. These response packets contained forged A and CNAME records pointing to malicious IP addresses. Simultaneously, we used the Scapy library to control packet fragmentation, predicting the values of the IPID field to achieve fragment rearrangement attacks.

We implemented the DNS-Sensor system in DNS Forwarders. The implementation involved establishing a cache database, monitoring DNS query requests and authoritative query responses, comparing the consistency of response data with cached data, and implementing a real-time alert mechanism.

We ran multiple experiments, simulating various types of fragment rearrangement attacks and observed the defense effectiveness of the DNS-Sensor system. Simultaneously, we recorded various data during the experiments, such as cache hit rates, alert frequencies, etc.

### 5.2. Experimental Results

Experimental results indicate that the DNS-Sensor system effectively defends against fragment rearrangement attacks on DNS Forwarders. Under simulated attacks, the DNS-Sensor system successfully detected and alerted on malicious response packets, halting their caching to safeguard DNS resolution.

The establishment and maintenance of the cache database were time-efficient, and the computational overhead for real-time monitoring and comparison processes was minimal.

In the validation experiments conducted on the regional authoritative mirror query system, we opted to examine the most recent data for the .com and .net top-level domains obtained from CZDS. The compressed .com zone file measured 4.1 GB, expanding to an uncompressed text file of 22 GB, encompassing 157,844,204 domains within the “.com” namespace. Similarly, the compressed .net zone file was 385 MB, expanding to an uncompressed text file of 1.9 GB, containing 13,494,414 domains under the “.net” domain.

**Figure 4:**
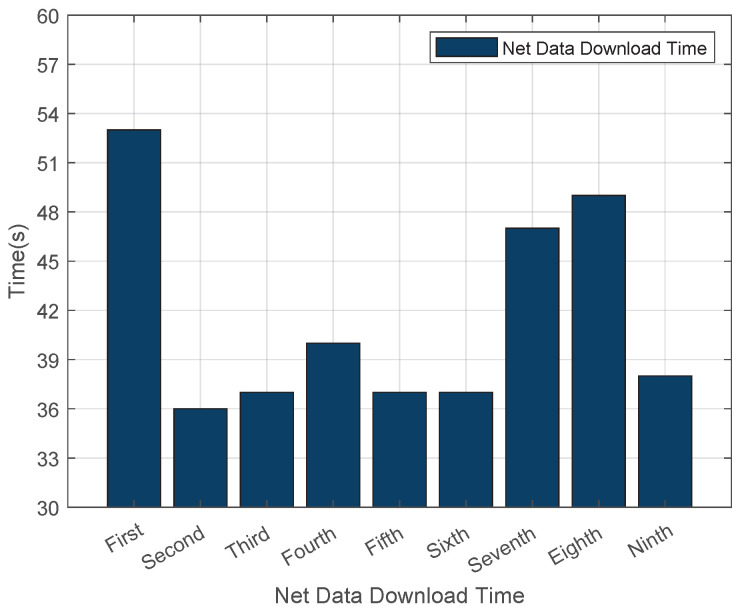
Download Time for Cold Data in the .net Zone.

**Figure 5:**
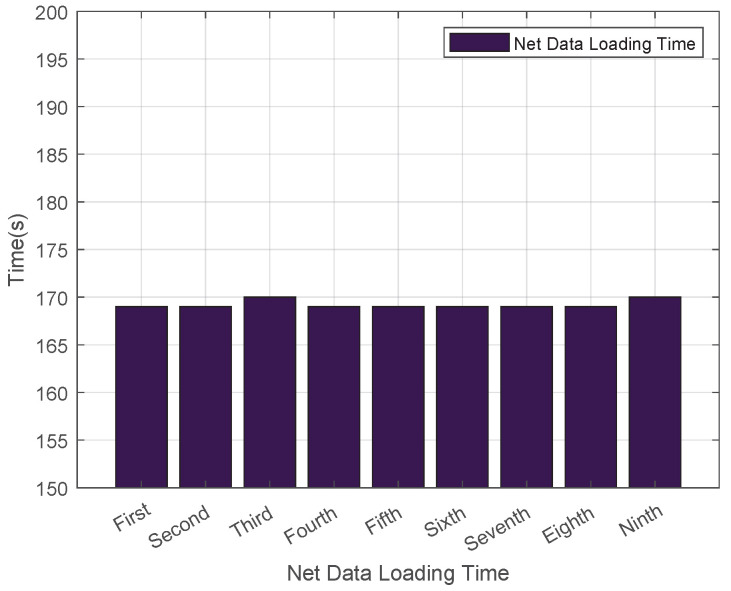
Load Time for Cold Data in the .net Zone.

**Figure 6:**
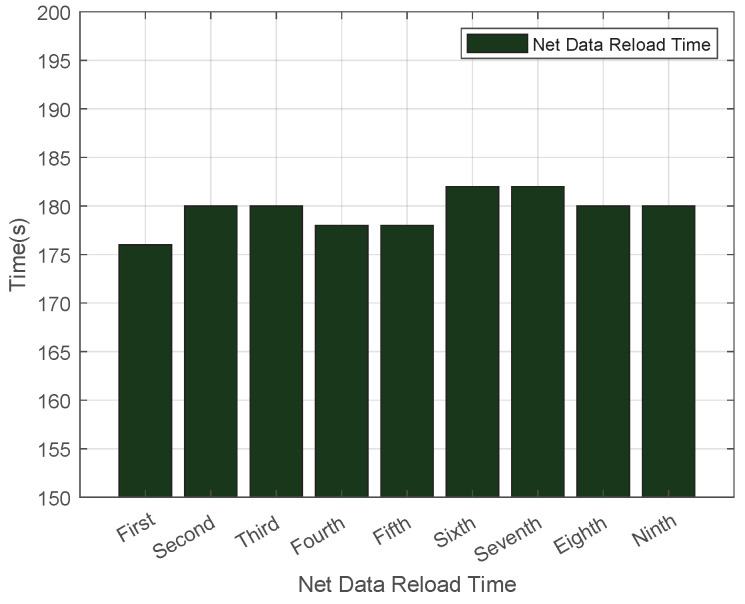
Reload Time in the .net Zone.

As illustrated in Figure 4, the average download time for .net domain data was recorded at 42 s. The fluctuation in download time is caused by changes in the network environment. This setup aligns with the actual scenario of DNS zone file updates in real networks, and the fluctuating data can more truly reflect the download speed of the system in a normal network. We conducted multiple experiments, and the results showed high stability. Figure 5 showcases the outcomes of multiple load tests on .net top-level domain files using Bind9, which revealed that the average cold data loading duration for the .net domain reached 170 s. Additionally, Figure 6 demonstrates that the average reload time for .net zone files amounted to 180 s. Such consistent results across multiple experiments indicate that our experimental data is highly stable.

**Figure 7:**
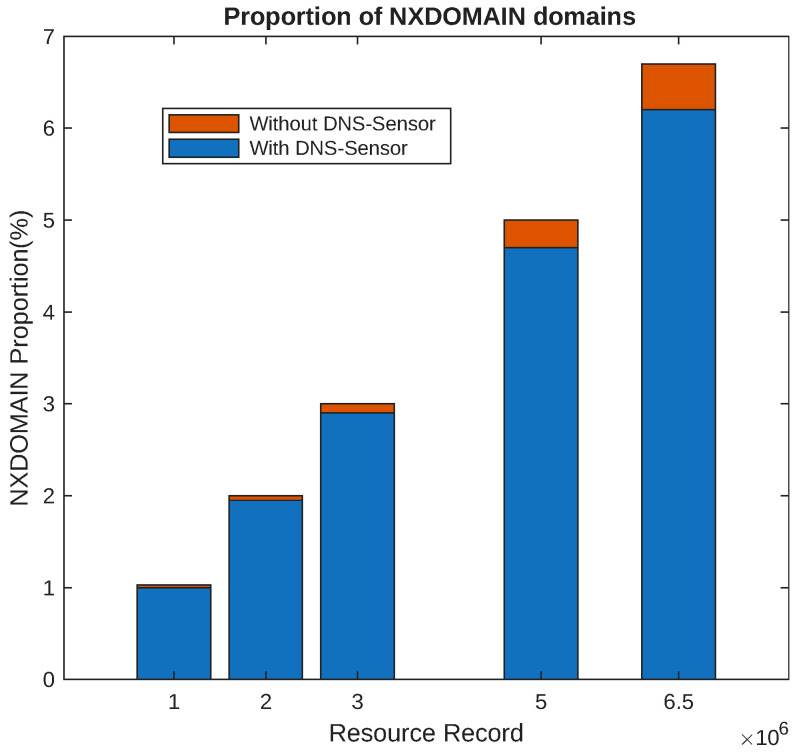
The proportion of NXDOMAIN domains.

Experimental findings indicated that the DNS-Sensor system exhibits high levels of flexibility and scalability. It can select diverse comparison methods and data storage strategies according to actual scenarios, with corresponding adjustments made to the alert mechanism. Comparison with Other Methods: A comparative analysis was carried out between the DNS-Sensor system and other commonly used DNS cache poisoning defense methods. As shown in Figure 7, when the number of resolution records in the cache increases, on the one hand, the proportion of NXDOMAIN domains increases both with and without the DNS-Sensor. When using the DNS-Sensor, we can clearly observe that the proportion of NXDOMAIN domains is less than that without using the DNS-Sensor. Meanwhile, as the number of resolution records in the cache increases, the effect of the DNS-Sensor becomes more obvious, and the reduction in the proportion of NXDOMAIN domains brought by using the DNS-Sensor is more significant. As presented in Table 2, experimental data verified that the DNS-Sensor system not only surpasses other methods in defending against fragment rearrangement attacks but also maintains a certain edge in overall performance metrics.

## 6. Conclusions

This paper conducts research on DNS cache attacks, analyzing major attack methods such as DDoS attacks, fragment rearrangement attacks on DNS forwarders, and DNS server hijacking. These attacks pose severe threats to the security and stability of the Internet by depleting resources, manipulating data packets, and tampering with response data. To counter these threats, this paper proposes a defensive strategy based on the DNS-Sensor system. This system can promptly detect and prevent the caching of malicious response data by comparing cached data with authoritative data in real-time, ensuring the security of DNS resolution. For different types of attacks, corresponding measures are adopted: for DDoS attacks, robust defense measures are deployed on DNS servers, coupled with collaboration with service providers such as CDNs to ensure service stability; and for fragment rearrangement attacks on forwarders and server hijacking, server security management is strengthened, including updating and maintaining software, restricting external access, and preventing intrusion and data tampering.

Experiments show that the DNS-Sensor system has effective defense capabilities and good performance, but further research and optimization are still needed in terms of practical application effectiveness and performance, and this research has certain limitations. For example, regarding the security threats caused by DNS packet fragmentation, in theory, fragmentation can be avoided by strictly limiting the length of DNS messages. However, in practice, this solution has limited application scenarios due to constraints from Domain Name System Security Extensions (DNSSEC) and response payload sizes, and it is still necessary to combine other defense methods to deal with fragmentation-related attacks. In addition, DNS cache attacks involve multiple components and stages, including DNS servers, Forwarders, recursive resolvers, etc., so defense requires collaborative efforts from multiple parties.

Nevertheless, many Forwarders, especially those on devices like home routers, have weak functionality and limited computing power. To address this, methods such as simplifying comparison logic, shortening cache time, utilizing auxiliary servers, and selecting more appropriate devices can be adopted to balance the relationship between security and device resources.

Future research will focus on multiple aspects: exploring more cache consistency check mechanisms, introducing technologies such as machine learning to improve detection accuracy and efficiency, and studying other types of DNS cache poisoning attacks to build a more comprehensive DNS security protection system; delving into exploring and improving defense mechanisms to cope with new threats arising from the continuous evolution of attack techniques; optimizing cache consistency verification mechanisms, expanding attack sample libraries to improve defense models against new types of cache poisoning methods, constructing a cross-component collaborative defense framework, promoting data sharing and linkage response among various components, and forming a global protection network.

As attack techniques continue to evolve, DNS security defense needs to maintain dynamic iteration. Through continuous efforts, technological innovation, and ecological collaboration, the DNS-Sensor system is expected to make greater contributions to ensuring the security and stability of DNS resolution, effectively enhancing the security of the DNS system, and guaranteeing the normal operation of the Internet and the security of user data.

## Figures and Tables

**Table 1:** Comparison of Common Methods for Cache Consistency Checks.

Check Method	Advantages	Disadvantages
Hash Verification	High-efficiency detection with ensured data integrity and tamper identification capability	Ineffective when subtle differences exist in resolved record results. Potential for hash collisions, leading to misjudgments.
Record Comparison	Increased computational overhead versus hash verification, offset by superior accuracy	Requires resolution of each queried domain’s results one by one, resulting in significant resource consumption.
Blockchain Technology	Immutable and decentralized, providing highly trusted data storage and verification. Real-time recording and verification of cache data to prevent tampering.	Introduces complexity and resource overhead, including the cost of consensus algorithm computation and blockchain maintenance. May lead to capacity and speed issues for frequent DNS queries.
Redundant Data Comparison	Increased trustworthiness of cache consistency through data comparison across multiple independent cache nodes. No reliance on complex algorithms, easy to implement and deploy.	Requires maintenance of multiple independent cache nodes, increasing resource and management costs. For large-scale systems, may lead to network latency and data synchronization issues.

**Table 2:** Improvement in Authoritative Resolution Time by DNS-Sensor system.

Item	Normal Resolution Success Rate (%)	Disaster Recovery Resolution Success Rate (%)	Normal Authoritative Average Resolution Time (ms)	Disaster Recovery Authoritative Average Resolution Time (ms)
legitimate 1000 Domains	100	100	15.055	14.807
Random 1000 Domains	99.9	100	68.819	5.232

## Data Availability

The research data comes from real DNS resolution data, which contains some user privacy and is not convenient to be made public.
